# Non-Traditional Antibacterial Agents Relevant to the WHO 2024 Priority Pathogens: A Structured Update of the Clinical Pipeline

**DOI:** 10.3390/antibiotics15090899

**Published:** 2026-09-12

**Authors:** Melanie Kempf Hansen, Lars Østergaard

**Affiliations:** Aarhus University Hospital (AUH), 8200 Aarhus, Denmark; lars.ostergaard@clin.au.dk

**Keywords:** antimicrobial resistance, non-traditional antibacterials, antibiotics, bacteriophage therapy, monoclonal antibodies, antimicrobial peptides, WHO priority pathogens, live biotherapeutic products

## Abstract

Background/Objectives: Antimicrobial resistance (AMR) is an urgent global threat, with bacteria developing resistance against most antibiotics currently in clinical use. Non-traditional antibacterials—including bacteriophages, antimicrobial peptides (AMPs), monoclonal antibodies (mAbs), live biotherapeutic products (LBPs) and anti-virulence small molecules—act through mechanisms distinct from traditional antibiotics and may exhibit mechanism-dependent differences in the propensity for resistance development. Despite growing interest, the landscape of non-traditional candidates in active clinical development against WHO priority pathogens has not been recently systematically mapped. This review aimed to characterize the current non-traditional antibacterial clinical pipeline targeting pathogens on the WHO Bacterial Priority Pathogens List (BPPL) 2024. Methods: Candidates were identified from the WHO antibacterial clinical pipeline report (October 2025) and verified through the Global AMR R&D Hub, ClinicalTrials.gov, and targeted searches of peer-reviewed publications, conference abstracts, and company communications. Candidates were included if they showed documented clinical activity since January 2023. Results: Twenty-seven candidates met the inclusion criteria, of which 20 remain in active clinical development and seven have been discontinued or paused or have unverified status. The pipeline is dominated by bacteriophages and markedly skewed toward carbapenem-resistant *Pseudomonas aeruginosa* (CRPA) and methicillin-resistant *Staphylococcus aureus* (MRSA), with few or no candidates targeting carbapenem-resistant *Acinetobacter baumannii* (CRAB), Shigella or Salmonella. Conclusions: Non-traditional antibacterials represent a promising but underfunded and unevenly distributed pipeline—with public and philanthropic investment in antibacterial R&D totaling just $2.51 billion across 2017–2023, a seven-year total, against an estimated $251–276 billion in global pharmaceutical R&D spending in a single recent year. Realizing their potential will require substantially greater investment and more deliberate prioritization toward the WHO priority pathogens currently underserved by the non-traditional pipeline.

## 1. Introduction

**Figure 1:**
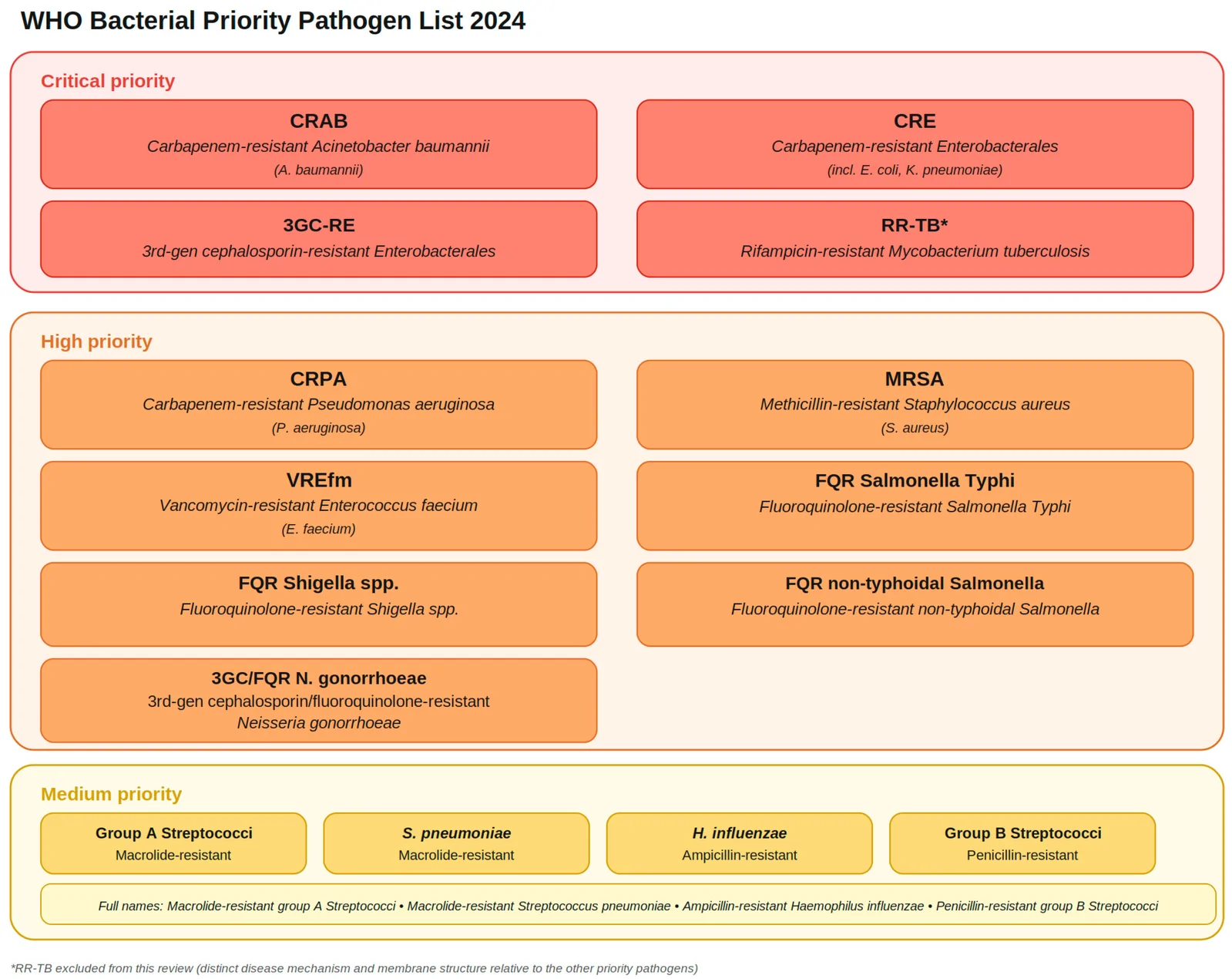
WHO Bacterial Priority Pathogen List 2024.

Antimicrobial resistance (AMR) is a major threat to public health. The continued use and misuse of antibiotics have led to an exponential rise in resistant pathogens, limiting treatment options. There is a global and urgent need for new strategies and drugs to treat multidrug-resistant (MDR) bacterial infections, a need recognized by many organizations, including the World Health Organization (WHO). In May 2024, WHO revised the Bacterial Priority Pathogens List to include 15 antibiotic-resistant priority pathogens and tackle the evolving challenges of AMR (Figure 1). The critical priority designation was given to carbapenem-resistant *Acinetobacter baumannii* (CRAB), carbapenem-resistant *Enterobacterales* (CRE), third-generation cephalosporin-resistant Enterobacterales and rifampicin-resistant *Mycobacterium tuberculosis*. Seven pathogens were given the high-priority designation, including methicillin-resistant *Staphylococcus aureus* (MRSA), vancomycin-resistant *Enterococcus faecium* (VREfm) and carbapenem-resistant *Pseudomonas aeruginosa* (CRPA). Four pathogens were given the medium-priority designation [1]. Bacteria develop resistance as a response to the antibiotics intended to target them, and the resulting mechanism of AMR depends on the specific antibiotic’s mechanism of action (MoA): upregulation of efflux pumps decreases drug accumulation in the bacterial cell, downregulation of cell wall porins limits drug uptake, upregulation of certain enzymes such as beta-lactamases inactivates the drug and lastly bacteria can also modify the drug target itself, through chemical modification or enzymatic degradation of its structure, rendering the drug ineffective [2,3].

AMR is a leading cause of death worldwide. In 2021, approximately 4.71 million deaths were associated with AMR and ~25% of these deaths were directly attributable to antibiotic-resistant pathogens. Of both deaths associated with and attributable to AMR, MRSA-associated deaths increased the most globally from 1990 to 2021. Projections suggest 8.22 million deaths will be associated with AMR globally in 2050 and that 1.91 million of these deaths will be directly attributable to AMR [4].

Beyond these population-level statistics, AMR also reshapes care for the individual patient: it harms patients by making common infections harder and sometimes impossible to treat, leading to prolonged illness, longer hospital stays and a higher risk of severe complications like sepsis. Patients often require second- and third-line antibiotics, which can cause severe side effects like organ failure, and in critical cases doctors are left with no effective treatment options, increasing the risk of death. As a result, routine treatments that rely on antibiotics, such as organ transplants, joint replacements and C-sections, become more dangerous to perform [5,6].

The economic value assigned to antibiotics does not reflect the benefits they bring. Almost all antibiotic research is now conducted by small biotech companies or academics. Public and philanthropic investment in antibacterial R&D totaled just $2.51 billion across 2017–2023, a seven-year total, against an estimated $251–276 billion in global pharmaceutical R&D spending in a single recent year [7,8], illustrating the chronic underinvestment in this area. This funding gap is reflected in the pipeline itself: only five novel classes of antibiotics have been marketed since 2000—oxazolidinones, lipopeptides, pleuromutilins, tiacumicins and diarylquinolines [9].

As highlighted in the WHO analysis of antibacterial agents in preclinical and clinical development in 2025, the clinical pipeline is still dominated by traditional antibacterial agents (50/90), most of which are multiple analogs of existing classes, especially beta-lactamase inhibitor combinations. The potential for rapid resistance development creates an additional challenge for antibiotic development, and is itself an argument for non-traditional antibacterials, which may exhibit mechanism-dependent differences in the propensity for resistance development, though clinical evidence remains insufficient to establish a consistently lower resistance risk.

Non-traditional antibacterials are defined here as agents that act through mechanisms fundamentally distinct from classical antibiotics—they do not inhibit conserved bacterial biosynthetic targets such as cell wall synthesis, protein synthesis and DNA replication. This encompasses a diverse range of non-traditional antimicrobial approaches: bacteriophages that exploit bacterial surface receptor specificity to achieve direct killing; antimicrobial peptides that disrupt membrane integrity through physical mechanisms; monoclonal antibodies that neutralize virulence factors or biofilm components; live biotherapeutic products that restore colonization resistance through microbial competition; and small-molecule and anti-virulence agents that disable bacterial pathogenicity [10,11]. What unites these otherwise heterogeneous approaches is that they do not rely on the same bacterial targets that existing resistance mechanisms are optimized against—offering, in principle, a route around established resistance genotypes.

These approaches represent a promising and increasingly important complement to traditional antibiotic development. The risk in new antibiotic development remains high, since resistance can emerge very fast after a new drug enters the market, and sometimes even during clinical trials [12].

Several recent reviews have characterized parts of this landscape: Blaskovich and Cooper provide a timely overview of emerging antimicrobial classes [13], Paterson and colleagues have addressed the Gram-negative pipeline specifically [14], and Butler et al. examine drug classes with novel mechanisms more broadly [15]. However, a candidate-level analysis of the non-traditional clinical pipeline mapped specifically to the WHO 2024 BPPL priority pathogens, and incorporating the most recent Phase II and Phase III candidates, has not yet been performed.

The WHO antibacterial pipeline report (October 2025) provides the most recent institutional overview of the field, but does not provide individual candidate annotations, updated development status as of mid-2026, or mapping to the WHO 2024 BPPL at the candidate level. This review provides a structured update of that pipeline snapshot.

This article aims to fill that gap as a structured pipeline review of the current non-traditional clinical antibacterial pipeline, with individual candidate annotations. It examines the current landscape of non-traditional antibacterials against these priority pathogens, highlighting their advantages and limitations and describing their mechanisms of action, while making the case for why they may represent a way forward in this neglected area of antibiotic development. This review identifies 27 candidates with documented activity since January 2023 (Table 1), reveals a pipeline dominated by bacteriophages and markedly skewed toward CRPA and MRSA, and highlights substantial gaps against several WHO priority pathogens.

## 2. Results

To facilitate interpretation of the candidate entries that follow, each candidate is assigned one of three pathogen indication categories, stated in parentheses after the candidate’s name together with its evidence level. Category A: clinical trials require patients to have the specific WHO-defined resistant phenotype (e.g., confirmed VRE infection). Category B: trials target the bacterial species but do not require the specific resistant phenotype. Category C: the link to the resistant pathogen is supported by preclinical or in vitro data only. The evidence level (L1–L5) reflects the strongest publicly available evidence as of the last search date; full definitions are provided in Section 4 (Methods), and all assignments are listed in Table 1 and Supplementary Table S1.

### 2.1. AMPs

Antimicrobial peptides are a class of naturally occurring small-molecule peptides, typically with a length of 12–50 amino acids, which act as a first line of defense against microbes. AMPs exist widely in nature and have been found in many different species like bacteria, plants, birds and fish [16]. AMPs are a diverse group of molecules and vary in their chemical structures and amino acid compositions, and are typically divided into natural and synthetic peptides. As of June 2026, 5512 antimicrobial peptides were identified, according to one database (APD; https://aps.unmc.edu (accessed on 9 June 2026)) [17]. This dataset includes both natural and synthetic AMPs. AMPs use two different mechanisms of action in order to kill bacteria, (1) membrane targeting or (2) intracellular targeting. AMPs cause physical membrane destruction by permeabilizing the bacterial cell membranes, either by inserting into the membrane and creating pores (barrel-stave or toroidal pore model) or by covering the membrane in peptides (carpet model). Membrane disruption leads to leakage of ions and intracellular metabolites, causing the membrane to depolarize and ultimately leading to cell lysis [18]. Some AMPs can enter the cytoplasm of bacteria and target cellular processes essential for pathogen survival, including the inhibition of DNA replication, protein synthesis, and interference with nucleic acid biosynthesis, metabolism, cell division, cell wall and LPS binding proteins—which is also the reason that some frameworks group AMPs as a traditional antibacterial [19].

AMPs have been shown to inhibit the production of biofilm by *S. aureus*, and have many attractive features of a novel antimicrobial class, a broad activity spectrum, a reportedly low incidence of bacterial resistance and a specific membrane-targeting mode of action (pore formation). Most clinically advanced AMPs today have been developed for topical applications [20]. Natural AMPs are sensitive to degradation and inactivation by proteases, and thus many synthetic AMPs are modified with the aim of making them more stable against this degradation. Limitations include more the expensive production cost of peptides versus DNA/RNA and that AMPs of nonhuman origin may be toxic to human cells [20].

#### AMPs in the Clinical Pipeline

OMN6 (Category B; L4) is a synthetic cyclic analog of the host defense peptide, cecropin A, naturally produced by the moth *Hyalophora cecropia*. OMN6 was engineered to target the negatively charged outer membrane of Gram-negative bacteria. Unlike cecropin A, OMN6 is stabilized through disulfide bond cyclisation, conferring resistance to proteolytic degradation—often a limitation for AMPs—while preserving bactericidal activity. OMN6 disrupts bacterial membrane integrity through electrostatic attraction to the negatively charged lipid head groups of the membrane phospholipids, leading to membrane depolarization and cell lysis, independently of bacterial resistance-genotype, including carbapenem- and colistin-resistant strains [21,22]. In murine models of MDR *A. baumannii* lung and bloodstream infections, OMN6 demonstrated a significant reduction in bacterial burden in preclinical studies [22]. The Phase I trial demonstrated that OMN6 was safe and well-tolerated in healthy adults, with no serious adverse events, as reported by the company [23]. The Phase II trial (NCT06087536) initiated dosing in June 2026, as reported by the company, representing the most clinically advanced systemic AMP candidate currently in active development for CRAB infections [24].

PLG0206 (Category B/C; L2) is a synthetic 24-amino acid engineered cationic AMP (eCAP), computationally optimized to contain only arginine, valine, and tryptophan residues to maximize bacterial membrane binding while minimizing host cell toxicity. Unlike natural AMPs, PLG0206 was specifically designed to penetrate and eradicate biofilm on prosthetic implants, a clinical unmet need, as biofilm-associated *S. aureus* is the leading cause of prosthetic joint infection (PJI), and current antibiotics fail to penetrate the biofilm matrix. PLG0206 disrupts bacterial membranes through electrostatic interaction with negatively charged lipid bilayers, targeting both planktonic and biofilm-embedded persister cells regardless of antibiotic resistance profile, with demonstrated broad-spectrum in vitro activity against MRSA, CRPA, CRE, VRE, and CRAB, as reported in peer-reviewed publications [25,26]. The interaction of PLG0206 with the membrane leads to lipid-phase consolidation, resulting in localized stiffening and ultimately membrane disruption [27]. In the completed Phase 1b trial (NCT05137314), PLG0206 demonstrated a favorable safety and pharmacokinetic profile, as reported in a peer-reviewed publication [28]. The pivotal Phase II/III RETAIN trial (NCT07214311) enrolled its first patients in March 2026, as reported by the company [29], evaluating PLG0206 as an intra-articular adjunct to DAIR (Debridement, Antibiotics and Implant Retention) surgery in patients with *S. aureus* PJI. A notable limitation is that PLG0206 exhibited >50% binding to a subset of human receptors, channels, and transporters at 10 μM in safety pharmacology screening, as reported in a peer-reviewed publication. The functional significance of these interactions remains under evaluation, as reported in a peer-reviewed publication [27]. Category B represents MRSA indication; Category C represents CRPA, CRE, VRE and CRAB, where activity is based on in vitro data only.

PL-5 (peceleganan) (Category C; L1) is a synthetic cationic AMP delivered as a topical spray targeting the negatively charged lipid bilayer of Gram-negative and Gram-positive bacteria, including MDR strains, with comparable activity against resistant and susceptible isolates [30]. Phase IIb (ChiCTR2000033334, *n* = 220) studied the efficacy and safety in patients with mild infections of diabetic foot ulcers and Phase III (ChiCTR2100047202, *n* = 570) randomized, open, active-controlled trials conducted in China demonstrated statistically superior clinical efficacy over silver sulfadiazine in patients with skin infections, with no severe adverse events, as reported in peer-reviewed publications [31], making PL-5 the most clinically advanced AMP by trial stage. Development and regulatory submission are currently focused on China, and the topical route precludes use in invasive MDR infections. It has yet to be tested on serious infections in clinical settings [30,31]. A phase II study (NCT06189638, *n* = 90) is currently active in the US, as registered on ClinicalTrials.gov, evaluating the efficacy and safety of PL-5 in mild infections of diabetic foot ulcers.

### 2.2. Bacteriophages in the Clinical Pipeline

Bacteriophages are bacteria-targeting viruses that use bacteria as reproduction factories, ultimately leading to bacterial cell lysis in the process. This occurs through several steps: attachment, where bacteriophages recognize and attach to specific bacterial surface receptors; penetration, where genetic material is injected into the bacterial cell; biosynthesis, where the bacterial replication machinery is hijacked and viral components are produced; assembly, where the newly created viral components are assembled into mature bacteriophages; and lysis, where phage-produced enzymes break down the bacterial cell wall, resulting in the release of new phages [32].

Phages are diverse and highly selective in the bacteria they target. The tips of the tail structure bind with high specificity to structures on the bacterial cell surface. Phages attach to essential bacterial surface structures like capsule proteins, efflux pumps or type IV pili. Bacteria can become less susceptible or resistant to bacteriophages and phages are often administered as cocktails to mitigate this resistance challenge. When bacteria become resistant to phages, this is not without a fitness cost. If they alter their efflux pumps, they might be resensitized to antimicrobials to which they were previously resistant. If they alter capsid proteins, capsule formation may be reduced [33]. In recent years, phage therapy has become recognized as a promising approach to the challenges of AMR and is also the non-traditional antibacterial category with the most assets/drugs currently undergoing clinical trials targeting WHO-priority pathogens.

#### 2.2.1. Bacteriophages Targeting MRSA

AP-SA02 (Category B; L3) is a fixed multi-phage cocktail targeting *S. aureus* surface receptors and is being evaluated as an adjunct to the best available therapy in patients with complicated *S. aureus* bacteremia, including MRSA. In the Phase Ib/IIa randomized disarm trial (NCT05184764, *n* = 42), AP-SA02 demonstrated a significant improvement in clinical response at day 12–88% in the AP-SA02 arm versus 58% in the placebo arm (*p* = 0.047), with no patients in the AP-SA02 group experiencing non-response or relapse compared to 25% in the placebo group, as reported in a conference abstract and company pipeline page [34,35]. MRSA was the causative pathogen in approximately 38% of both the AP-SA02 and placebo groups. All MRSA/MSSA-infected subjects treated with AP-SA02 cleared their infection with no relapses through the end of the study. The cocktail incorporates defined phage variants, providing coverage of patient-to-patient variability in bacterial surface receptor expression—an intrinsic adaptive mechanism that broadens clinical applicability without requiring personalized phage selection [34]. Persistence of AP-SA02 in the intravenous (IV) compartment for multiple days post-infusion was demonstrated, and clinical responses observed across diverse infection sites—including septic joint, osteomyelitis, and endocarditis—suggest phage distribution and presumed biofilm penetration, consistent with known phage biology. AP-SA02 has only been evaluated as an adjunctive therapy and not as monotherapy. AP-SA02 has received U.S Food and Drug Administration (FDA) Fast-Track designation, and a Phase III pivotal trial is planned for initiation in 2026, as reported by the company [35].

BX211 (Category B; L3) is a personalized natural phage cocktail targeting *S. aureus*, including MRSA, delivered as a local adjunct to standard-of-care systemic antibiotics therapy in patients with diabetic foot infections (DFI), to prevent progression to osteomyelitis (DFO). The Phase 2 DANCE trial enrolled patients with established DFO, and in this trial BX211 was associated with a statistically significant and sustained reduction in ulcer size from Week 7 onwards, with a difference exceeding 40% by Week 10 (*p* = 0.046 at Week 12), as reported in a conference abstract [36]. The local delivery limits its use to accessible infection sites, and the personalized approach requires individual phage selection. Following FDA feedback, development has transitioned from BX211 to a fixed multi-phage cocktail termed BX011. This new cocktail incorporates phages evaluated in the DANCE trial, and a Phase II/III study with BX011 is planned but not yet registered [37].

PP1493 and PP1815 (Category B; L3) form a natural two-phage cocktail, targeting *S. aureus* in biofilm-associated PJI, administered as intra-articular injections as an adjunct to DAIR surgery. Clinical proof-of-concept was established in three compassionate-use patients with recurrent prosthetic knee infections, and improved clinical condition was observed following intra-articular phage cocktail administration, as reported in a peer-reviewed publication describing compassionate use cases [38]. The phages belong to the Silviavirus and Rosenblumvirus genera, selected to cover a broad range of clinical *S. aureus* isolates through complementary receptor binding specificity. The Phase II GLORIA trial (NCT06605651) is currently recruiting globally, as confirmed by the trial registry, representing the first international randomized controlled trial of phage therapy for PJI. The local intra-articular delivery limits its use to accessible joint infections.

#### 2.2.2. Bacteriophages Targeting CRPA

Three inhaled phage programs targeting chronic pulmonary CRPA have now completed Phase I/II trials; the were results recently reviewed by Chan et al. [39].

AP-PA02 (Category B; L2) is a natural multi-phage cocktail, consisting of bacteriophages from multiple lineages targeting multiple receptor classes, administered by nebulization as an adjunct to standard-of-care antibiotics for chronic pulmonary *P. aeruginosa* infection. In the phase Ib/IIa SWARMPa trial (NCT04596319, *n* = 29) in cystic fibrosis patients, high phage potency, persistence in the lung, and limited systemic distribution of AP-PA02 were demonstrated. In the Phase IIb Tailwind trial (NCT05616221, *n* = 48) in non-cystic fibrosis bronchiectasis (NCFB) patients, the pre-specified per-cohort (Cohort A = monotherapy; Cohort B = combination therapy) efficacy analysis was not statistically significant, which was attributed to the small cohort sizes. A post hoc combined intent-to-treat analysis showed a statistically significant reduction in *P. aeruginosa* colony forming units (CFUs) at Day 17 (*p* = 0.05) that persisted at Day 24 (*p* = 0.015), as reported in a peer-reviewed publication, though this was a post hoc analysis and not a pre-specified primary endpoint [40]. Inhaled delivery limits its use to pulmonary indications, and both cystic fibrosis (CF) and NCFB indications represent chronic colonization rather than acute CRPA infections. Armata Pharmaceuticals is planning a Phase III trial evaluating AP-PA02 as an alternative to antibiotics in chronic pulmonary *P. aeruginosa* infection, as reported by the company [40].

BX004-A (Category B; L2) is a three-phage cocktail designed to target a wide range of *P. aeruginosa* strains, administered through nebulization as an adjunct to standard of care antibiotics for chronic pulmonary infection management. In the Phase Ib/IIa trial (NCT05010577, *n* = 9), a reduction in the *P. aeruginosa* sputum burden was observed in the phage arm, with efficient delivery to the lower respiratory tract confirmed; the results were published in *Nature Communications* in 2025 [41]. The small sample size limits efficacy conclusions, and the indication was CF-focused rather than acute CRPA infection. The Phase IIb trial (NCT06998043), was on hold in the US due to FDA concerns with the third-party nebulizer device used for delivery, thought to be a temporary hold, as reported by the company [42], but was ultimately discontinued in December 2025 following a safety review [43].

YPT-01 (Category B; L2) is a personalized three-phage cocktail designed by Yale University, administered through nebulization as an adjunct to standard of care antibiotics for chronic pulmonary *P. aeruginosa* infection in CF patients. Personalized phage selection is based on individual patients’ isolate susceptibility and furthermore the phages are chosen to target distinct *P. aeruginosa* virulence structures. OMKO1 targets the efflux pump component OprM. TIVP-H6 targets type IV pili and LPS-5 targets the LPS O-antigen, as described in peer-reviewed publications [39]. This multi-target approach exploits an evolutionary trade-off—bacteria developing resistance to these must sacrifice key virulence factors, potentially lose pathogenicity and regain antibiotic susceptibility. The Phase Ib/II CYPHI trial (NCT04684641, *n* = 8) did not meet its primary endpoint despite evidence for decreased *P. aeruginosa* burden [39,44]. The personalized approach requires individual phage-susceptibility testing before treatment, adding logistical complexity and time.

WRAIR-PAM-CF1 (Category B; L3) is a four-phage cocktail (PaWRA01Phi11, PaWRA01Phi39, PaWRA02Phi83, PaWRA02Phi87, formerly designated WRAIR_EPa11, EPa39, EPa83, and EPa87) targeting *P. aeruginosa*, administered as a single IV infusion. The four phages were selected for complementarity of host range against clinical CF *P. aeruginosa* isolates and are free of lysogeny genes, antibiotic resistance genes, and toxin genes, as described in the published trial protocol [45]. The Phase Ib/II PHAGE trial (NCT05453578) is a multicenter, randomized, double-blind, placebo-controlled study enrolling up to 72 adult CF patients chronically colonized with *P. aeruginosa* across up to 20 US sites, with first patient dosing reported in January 2023 with IV delivery as an adjunct to standard-of-care antibiotics, as reported by the medical press [45,46]. Primary endpoints include safety and microbiological activity (reduction in pulmonary *P. aeruginosa* colony counts); secondary endpoints include pharmacokinetics and lung function [45]. No results have been published as of the most recent search.

TP-122A (Category B; L3) is a natural three-phage cocktail targeting *P. aeruginosa* and administered as an adjunct to standard-of-care antibiotics in patients with ventilator associated pneumonia (VAP). Delivered by nebulization to achieve direct pulmonary delivery, TP-122A constitutes the *P. aeruginosa*-targeting component of the broader TP-122 platform, which also includes TP-122B targeting *Klebsiella pneumoniae (K. pneumonia)*, reflecting a hospital-based phage therapy strategy against the major pathogens causing VAP [47]. A Phase I/IIa randomized, open-label trial (NCT06370598) (*n* = 15) is evaluating safety, tolerability, reduction in bacterial load, and clinical outcomes as primary and secondary endpoints (EU Clinical Trials 2025-521533-85-00). The trial’s estimated primary completion was May 2025; as of the most recent search, no results have been published. Given the small sample size, VAP-specific indication, and absence of published clinical data, TP-122A represents a pipeline candidate of uncertain current status rather than an established evidence-based program.

#### 2.2.3. CRISPR-Armed Bacteriophages

CRISPR-armed bacteriophages combine the natural host specificity of bacteriophages with a CRISPR-Cas nuclease system that is delivered into the bacterial cell upon phage infection [48,49]. Once inside the cell, the CRISPR-Cas system (Cas3 in both clinical-stage candidates described below) is guided to specific chromosomal sequences, where it cleaves and degraded the bacterial DNA, killing the cell independently of its antibiotic-resistance profile. This dual mechanism of phage lytic activity combined with targeted chromosomal DNA degradation confers killing efficacy against both antibiotic-susceptible and -resistant strains [49].

In the context of *Escherichia coli* (*E. coli*) gut decolonization, CRISPR-armed bacteriophages are administered orally, allowing them to reach the gastrointestinal tract where they selectively eliminate target *E. coli* strains while sparing the broader commensal microbiota, since the CRISPR-targeting sequences are designed to match only the targeted species [48]. For urinary tract infection, a combined intravenous and intraurethral route is used. Clinical evidence to date is early but encouraging: SNIPR-001 covered 90.3% of a clinically relevant *E. coli* panel in vitro and showed a favorable safety profile with a stable gut microbiome in Phase I [50], while LBP-EC01 produced a rapid *E. coli* reduction in urine within four hours of first dose administration in Phase II [51]. These findings are described for each candidate below.

SNIPR-001 (Category B; L2) is a cocktail of four Clustered Regularly Interspaced Short Palindromic Repeats (CRISPR)-armed bacteriophages designed for precision gut decolonization of antibiotic-resistant *E. coli*, including fluoroquinolone-resistant and MDR strains, while preserving the broader commensal microbiota and covering a broad range of *E. coli* strains. Through a dual mechanism, SNIPR-001 causes bacterial cell lysis through the bacteriophage lytic cycle and simultaneously injects CRISPR-Cas3 machinery targeting five specific chromosomal regions encoding virulence genes (*fimH*, *bolA*, *rpoH*) and essential genes (*lptA* and *murA*), leading to chromosomal DNA cleavage and bacterial cell death [48]. In vitro host range covers 90.3% of a relevant *E. coli* panel originating from blood samples drawn from patients with hematological cancer, as reported in a peer-reviewed publication [50]. The Phase I trial (NCT05277350, *n* = 36) demonstrated favorable safety and tolerability across three dose levels and stable gut microbiome composition [50]. The Phase Ib trial targeting hematological cancer patients undergoing HSCT initiated dosing in June 2025 and is currently more than halfway enrolled, co-funded by CARB-X [52]. FDA has granted Fast-Track designation for prophylaxis to prevent bloodstream *E. coli* infections in patients with hematological cancer at risk of neutropenia (low neutrophil count), as confirmed in a peer-reviewed publication [50]. The prevention-focused decolonization indication and lysis zone activity of uncertain clinical relevance remain key limitations [48].

LBP-EC01 (Category B; L2) is a six-phage CRISPR-armed bacteriophage cocktail, delivering enhanced killing efficacy over natural phages through chromosomal DNA degradation combined with phage lytic activity, conferring antibacterial activity independent of Antibiotic Resistance status. Unlike SNIPR-001, which targets gut *E. coli* colonization, LBP-EC01 is administered by combined IV and intraurethral delivery for the treatment of acute uncomplicated urinary tract infections (UTIs) caused by drug-resistant *E. coli*—one of the largest contributors to global antibiotic consumption. In part one of the two-part Phase II ELIMINATE trial (NCT05488340, *n* = 31), a rapid reduction in *E. coli* in the urine was observed by four hours after the first treatment, maintained at Day 10 for all 16 evaluable patients, as reported in a peer-reviewed publication [51]. Part two is currently enrolling. Development is supported by up to $93 million in Biomedical Advanced Research and Development Authority (BARDA) funding as part of a $152 million program supporting Phase II/III and potential marketing approval, as reported by the company [53]. Key limitations include the combined IV and intraurethral delivery route, which adds complexity, and the absence of confirmed resistance-phenotype enrollment criteria. The trial enrolled patients with broadly drug-resistant *E.coli* UTI.

#### 2.2.4. Other Bacteriophages

TP-102 (Category B; L2) is a natural five-phage polymicrobial cocktail targeting MRSA, CRPA and CRAB, distinguishing it from other phage cocktails in clinical development, which target a single pathogen. TP-102 is applied topically to infected diabetic foot ulcers (DFU) as an adjunct to standard wound care. The Phase I/IIa REVERSE trial (NCT04803708) demonstrated safety and tolerability with no significant adverse events when applied to infected and non-infected foot ulcers [54]. TP-102 holds FDA Fast-Track designation for DFU indication targeting three bacterial species—the broadest pathogen coverage of any phage cocktail in the clinical pipeline [55]. The Phase IIb REVERSE2 trial (NCT05948592) was completed in December 2025, with results pending peer-reviewed publication and Phase III preparation underway [56]. Limitations include topical-only delivery, restricting its use to accessible wound infections, and the requirement for prior susceptibility testing before treatment.

VRELysin (Category A; L4) is an oral bacteriophage cocktail targeting vancomycin-resistant *Enterococcus* (VRE) in the gastrointestinal tract—the only non-traditional candidate in the current clinical pipeline specifically targeting VRE, a WHO high-priority pathogen. Through the phage lytic killing of VRE in the gut, VRELysin aims to reduce or eliminate VRE colonization levels in the gastrointestinal tract, thereby preventing endogenous VRE bloodstream infections and nosocomial transmission. Phase I (NCT05715619, the same trial ID as Phase II) was completed in healthy volunteers, confirming its safety and tolerability. The Phase IIa study, designed to evaluate VRELysin in VRE-colonized patients, is currently paused due to a lack of funding, as confirmed by the trial registry, representing a significant development risk.

ShigActive (Category C; L2) is, to our knowledge, the only non-traditional drug in the current antibacterial clinical pipeline targeting fluoroquinolone-resistant *Shigella*—a WHO high-priority pathogen with an otherwise absent non-traditional pipeline. ShigActive is an oral natural multi-phage cocktail targeting *Shigella spp*. in the gastrointestinal tract, designed to prevent or reduce the severity of shigellosis. In the Phase I randomized double-blind controlled trial (NCT05182749, *n* = 10 healthy volunteers), ShigActive was safe and well tolerated with no significant adverse events, supporting advancement to a Phase IIa human challenge model study, as reported in a peer-reviewed publication [57]. The prevention-focused indication and human-challenge model design, rather than treatment of established clinical infection, reflect the early stage of development and the inherent challenges of conducting trials for enteric pathogens in high-burden settings. ShigActive represents an isolated example of non-traditional development for the neglected enteric pathogens on the WHO 2024 priority list—a gap that stands in stark contrast to the concentration of pipeline activity against CRPA and MRSA.

### 2.3. Live Biotherapeutic Products

Live biotherapeutic products (LBPs) are an emerging class of treatments utilizing live microorganisms to prevent or cure diseases. LBPs offer an alternative or a supplement to traditional antibiotics by restoring natural gut defenses to outcompete drug-resistant pathogens, and by utilizing engineered microbes to locally degrade antibiotics [58]. They are biological products that contain live microorganisms such as bacteria, which are applicable to the prevention, treatment or cure of a disease or condition [59].

#### LBPs in the Clinical Pipeline

SER-155 (Category B; L4) is an oral 16-strain microbiome therapeutic designed to restore gut microbial diversity, decolonize ESKAPE pathogens, including CRE and VRE, strengthen the intestinal epithelial barrier and prevent gut-derived bloodstream infections in patients undergoing allo hematopoietic stem cell transplant (HSCT) [60]. The Phase Ib trial (NCT04995653), with two cohorts, evaluated its safety, tolerability and efficacy in adults undergoing allo-HSCT. Cohort 1 biomarker analyses showed SER-155 was generally well tolerated through Day 100, with no drug-related serious adverse events, as reported in a conference abstract [61]. In the randomized double-blind placebo-controlled Cohort 2 (*n* = 34), SER-155 was associated with a significantly lower incidence of bacterial bloodstream infection compared to placebo (10% vs. 42.9%; *p* = 0.0423), a significantly shorter duration of systemic antibiotic treatment (9.2 vs. 21.2 days; *p* = 0.0494), and a lower incidence of febrile neutropenia (65% vs. 78.6%) [62]. No treatment-related serious adverse events were observed. These results supported FDA Breakthrough Therapy Designation, with a registrational Phase II in preparation, as reported by the company [62]. SER-155 is prevention-focused, targeting a specific immunocompromised population rather than treatment of established AMR infection.

### 2.4. Monoclonal Antibodies

Monoclonal antibodies (mAbs) are emerging as a vital tool in the combat against AMR. Unlike most traditional antibiotics that kill bacteria directly and also drive resistance, mAbs offer a targeted approach by neutralizing toxins, blocking bacteria from invading cells and tagging them for immune clearance without altering the hosts’ microbiota. Their high specificity causes narrow-spectrum applicability, raising concerns about their clear clinical benefits. Some current strategies are focusing on combination approaches or mAb cocktails, or generating antibodies against protein antigens conserved across multiple species. Treatment studies, including panobacumab, rivabazumab (in CF), tosatoxumab, and tefibazumab, showed variable clinical outcomes, with some signals of benefit in selected populations. However, these findings remain inconclusive, and no mAbs are currently used in clinical practice [63].

Beyond the challenges of narrow specificity and the need for rapid diagnostics, the clinical context of mAb deployment matters significantly—prophylactic use in well-defined high-risk cohorts differs fundamentally from treatment of established infection in terms of trial design and endpoint selection. The EVADE and SAATELLITE trials explored prophylactic mAb administration in mechanically ventilated patients colonized with *P. aeruginosa* and *S. aureus,* respectively, using the decrease in pneumonia incidence as the primary endpoint—an approach that has so far not demonstrated a statistically significant benefit in the overall trial population [63].

#### 2.4.1. Biofilm Disrupting Agents

CMTX-101 (Category B; L4) is a humanized mAb targeting the DNABII proteins; Integration Host Factor and Histone-like protein (IHF/HU), which are conserved DNA binding proteins critical for the structural integrity of the bacterial biofilm’s extracellular matrix. By disrupting the biofilm matrix, CMTX-101 exposes bacteria to host immune responses and concurrent antibiotics, thereby reducing the bacterial burden without direct bactericidal activity [64]. CMTX-101 shares these DNABII-targeting mechanisms with TLR1068 but differs by being more clinically advanced and specifically developed for chronic pulmonary *P. aeruginosa* infection. While the DNABII target is conserved across bacterial species, the current indication is narrow. In the phase Ib/IIa trial (NCT06159725, *n* = 17), 13 of 17 subjects achieved a greater than 70% reduction in *P. aeruginosa* CFU count at Day 28, with reductions in inflammatory biomarkers also reported by the company [65]. CMTX-101 received FDA Fast-Track designation in August 2025, as reported by the company [66]. The biofilm disruption mechanism requires concurrent antibiotics and host immunity, and the CF indication limits applicability to chronic pulmonary infection rather than acute CRPA.

TLR1068 (Category B; L2) is a fully human monoclonal antibody targeting DNABII proteins (IHF/HU) in the extracellular biofilm matrix, which are critical for the matrix integrity across both Gram-positive and Gram-negative bacteria. It targets a conserved epitope on the bacterial binding proteins in biofilm that is only exposed after cell death, with no homologs in the human proteome [67]. The Phase I trial (NCT04763759, *n* = 15) evaluated TLR1068 activity in patients with chronic prosthetic joint infection in the hip or knee with different infections, including MRSA. The trial demonstrated favorable pharmacokinetics, tolerability and preliminary evidence of biofilm disruption, and showed reduced infection recurrence compared to control patients, with no significant adverse events, as reported in a peer-reviewed publication [68]. A Phase II study (NCT06621251) is registered, designed to assess the efficacy of TLR1068 in combination with DAIR surgery for chronic PJI, to eliminate the need for two-stage exchange arthroplasty. TLR1068 shares its DNABII-targeting mechanism with CMTX-101 but targets MRSA-associated PJI rather than CRPA pulmonary infection, illustrating the broad-spectrum potential of the anti-DNABII approach. As with CMTX-101, the biofilm disruption mechanism requires concurrent antibiotics or host immune function rather than providing direct bactericidal activity.

RESP-X (Category B; L3), also known as INFEX702, is a humanized IgG4 mAb targeting PcrV—the tip protein of the *P. aeruginosa* type III Secretion System (T3SS). By blocking PcrV, RESP-X inhibits T3SS-mediated toxin translocation into host cells, reducing epithelial cell damage, neutrophil destruction and inflammatory response without direct bactericidal activity, thereby restoring the ability of host immune cells to control *P. aeruginosa* [69]. The Phase IIa trial (*n* = 9, no trial registration number publicly available) in NCFB patients with chronic *P. aeruginosa* infection met its primary objectives, demonstrating safety and tolerability with no severe adverse events. Target coverage was reported across all *P. aeruginosa* isolates from patient sputum, with all sequences known to bind RESP-X, as reported in the conference abstract (ERS) [69] and confirmed by the company [70]. As an anti-virulence agent RESP-X does not directly reduce *P. aeruginosa* burden, and the current indication is limited to chronic colonization in NCFB rather than acute CRPA infection.

#### 2.4.2. Anti-Virulence/Anti-Toxin

Tosatoxumab (AR-301) (Category B; L1) is a fully human IgG1 monoclonal antibody targeting *S. aureus* alpha toxin (alpha-hemolysin)—a pore-forming cytotoxin critical for virulence, host cell destruction, and immune evasion. By neutralizing alpha-toxins before pore-formation in host cell membranes, Tosatoxumab aims to preserve immune cell function and reduce tissue damage in *S. aureus* VAP [71]. In the Phase III randomized, double blind, placebo-controlled trial (NCT03816956, *n* = 174), the primary endpoint of clinical cure at Day 21 was not met in the overall population (*p* = 0.23). A numerical improvement in clinical cure rate was observed in the subgroup of patients over 65 years (*p* = 0.056 at Day 21; *p* = 0.025 at Day 28). The clinical significance of this subgroup finding remains uncertain given the failed primary endpoint and borderline *p*-values. Development is currently stalled pending a commercial partnership. Together with Suvratoxumab (NCT05331885) and 9MW1411 (NCT05339802), tosatoxumab represents the third independent anti-alpha toxin mAb program to face significant clinical challenges, raising questions about the validity of this target as a standalone adjunctive approach.

MEDI3902 (gremubamab) (Category B; L1/L3) is a bispecific IgG1 monoclonal antibody targeting both PcrV—the tip protein of the *P. aeruginosa* T3SS—and Psl—an exopolysaccharide of *P. aeruginosa* involved in colonization and biofilm formation—combining virulence neutralization with complement-mediated opsonophagocytic killing in a single molecule [72]. The Phase II EVADE trial (NCT02696902, *n* = 188) was a randomized double-blinded, placebo-controlled study in mechanically ventilated intensive care unit (ICU) patients tracheally colonized with *P. aeruginosa*. *P. aeruginosa* pneumonia was confirmed in 22.4% of MEDI3902 1500 mg recipients versus 18.1% of placebo recipients, and the trial did not meet its primary endpoint [73]. As a systemically administered IV antibody, gremubamab must reach the airway compartment by distribution from serum into epithelial lining fluid. Serum exposure appears to have been adequate: 80.6% of subjects achieved serum concentrations above the threshold associated with improved outcomes in animal models, as reported in a peer-reviewed publication [73]. The failure to meet the primary endpoint despite adequate systemic exposure suggests either that serum-to-airway penetration was insufficient to achieve effective (neutralizing and opsonizing) antibody concentrations at the site of colonization, or that a prophylactic strategy in an already colonized, critically ill population was the key limiting factor; the trial was also stopped early due to low recruitment and was underpowered to detect small treatment effects [73]. Development was discontinued. Together with AR-105 and the anti-alpha toxin programs against *S. aureus*, MEDI3902 represents a third failed CRPA mAb program, suggesting that mAb-mediated virulence neutralization and opsonization in mechanically ventilated ICU patients faces translation challenges beyond pharmacokinetics.

However, clinical development of gremubamab continued in a different indication. The Phase II GREAT-2 trial (ISRCTN70034823, *n* = 37) evaluated gremubamab in patients with bronchiectasis and chronic *P. aeruginosa* infection, randomizing participants 1:1:1 to 500 mg, 1500 mg or placebo administered IV. Results presented at ATS 2025 and ERS 2025 showed a reduction in sputum *P. aeruginosa* load at Day 84 in the 500 mg arm compared to placebo (*p* = 0.071, meeting the trial primary endpoint [74]. AstraZeneca is now progressing AZD0292 (NCT07088926), a half-life extended version of gremubamab for the bronchiectasis indication [75], as reported by the company [76]. MEDI3902 (gremubamab) therefore represents an important illustration of the mechanism-over-modality argument. Its bispecific biofilm/virulence-targeting mechanism showed clinical signal in a chronic colonization context despite failing in acute prophylaxis—consistent with the pattern observed across CMTX-101 and RESP-X, although whether this reflects the mechanism or clinical setting cannot be resolved with the current data. L1 represents the EVADE peer-reviewed trial and L3 represents the GREAT-2 conference abstract.

### 2.5. Small- and Other Molecules in the Clinical Pipeline

CAL02 (Category C; L1) is a broad-spectrum anti-toxin liposomal nanoparticle consisting of empty unilamellar liposomes that act as competitive decoys for bacterial toxins produced by diverse pathogens including *Streptococcus pneumoniae* (*S. pneumonia*), MRSA, *K. pneumoniae* and *P. aeruginosa* causing severe pneumonia. Bacterial toxins insert into the liposome membrane rather than host cell membranes, neutralizing their cytotoxic activity and reducing inflammation and immune suppression [77]. The mechanism is pathogen-agnostic—active regardless of antibiotic-resistance genotype and before pathogen identification. In the Phase I randomized double-blinded placebo-controlled trial (NCT02583373, *n* = 19) in severe pneumococcal pneumonia, CAL02 was safe and well tolerated, with exploratory efficacy analyses suggesting improvement over placebo across assessed parameters in this small first-in-human trial, as reported in a peer-reviewed publication [77]. The Phase II trial (NCT05776004) is currently active. CAL02 has no direct antibacterial activity and is entirely dependent on concurrent antibiotics, and the indication is pneumonia broadly rather than a specific WHO priority pathogen.

GSK3882347 (Category C; L3) is a small-molecule FimH antagonist targeting the adhesin protein FimH on type I pili of uropathogenic *E. coli*. By competitively antagonizing FimH binding to mannose-rich glycoproteins on bladder epithelium such as uroplakin Ia, GSK3882347 prevents *E. coli* from attaching to the bladder wall, blocking the initial colonization step required for UTI establishment. GSK3882347 does not have bactericidal activity but disarms the bacteria without affecting the broader microbiome and in principle, without imposing direct selection pressure for resistance [78]. The Phase Ib trial (NCT05138822) is evaluating microbiological response at test-of-cure following five days of oral dosing in adult females with uncomplicated UTI. Efficacy is dependent on FimH expression, limiting coverage to *E. coli*-caused UTI’s—approximately 85% of cases—and excluding non-FimH-expressing uropathogens.

ALS-4 (Category C; L4) is an orally administered small-molecule anti-virulence agent targeting *S. aureus*, including MRSA, developed by Aptorum Group. ALS-4 inhibits dehydrosqualene desaturase (CrtN), the enzyme responsible for staphyloxanthin biosynthesis. Staphyloxanthin is a carotenoid pigment that protects *S. aureus* from reactive oxygen species (ROS) deployed by host neutrophils and phagocytic cells; by blocking its production, ALS-4 is intended to strip this protection and restore host’s innate immune killing capacity, without a human homolog target for CrtN [79]. In a completed Phase I randomized, double-blind, placebo-controlled single- and multiple-ascending-dose trial (NCT05274802, *n* = 72 healthy volunteers), ALS-4 was well tolerated with no serious adverse events and no clinically relevant changes in vital signs, ECG, or laboratory parameters, as reported in a company investor presentation [79]. Following a Pre-IND meeting with the US FDA in March 2023 the company reported a focus on Phase II development targeting acute bacterial skin and skin structure infections (ABSSSI) [80], though no Phase II trial had been initiated as of the most recent available information. As an anti-virulence agent, ALS-4 does not directly reduce bacterial burden and relies on intact host immune function for efficacy; its development beyond Phase I has not been independently confirmed through trial registry updates.

Rhu-pGSN (Category C; L5) is a recombinant human plasma gelsolin (pGSN) protein-replacement therapy. Unlike pathogen-targeted candidates, rhu-pGSN is host-directed and not pathogen-specific. It addresses the depletion of plasma gelsolin that occurs during serious infection and acute inflammatory states, restoring a protein that enhances macrophage uptake and the killing of bacterial pathogens [81]. An earlier completed Phase Ib/IIa trial (NCT03466073, NCT04358406) generated clinical data relevant to MDR CRPA, supporting the rationale for gelsolin repletion as an adjunct in serious Gram-negative infection, as reported in a peer-reviewed paper. As of the most recent search, rhu-pGSN’s most clinically advanced and actively recruiting program is a global Phase IIb trial (NCT05947955) evaluating its efficacy in acute respiratory distress syndrome (ARDS) caused by infection. It is delivered via IV to hospitalized patients with survival with or without organ failure at Day 28 as the primary efficacy measure, as reported by the company [82]. This trial is not pathogen-specific and represents a broader inflammatory-disease indication rather than a targeted AMR application. Its inclusion here reflects the mechanisms relevance to host-directed approaches against MDR pathogens rather than confirmation of an active, pathogen-targeted clinical program against any single WHO priority pathogen. 

AR-501 (Panaecin) (Category B; L4) is an inhaled gallium citrate solution developed by Aridis Pharmaceuticals, designed as a non-antibiotic, iron-analog anti-infective for chronic pulmonary CRPA infection in CF patients. Gallium mimics iron and is taken up by bacteria via iron-acquisition pathways but cannot substitute for iron in essential redox reactions, disrupting multiple iron-dependent bacterial processes simultaneously [83]. This multi-target mechanism is proposed to confer a low intrinsic resistance profile, since bacteria cannot easily evade iron deprivation across several pathways at once, and AR-501 is reported to act synergistically with co-administered antibiotics. Delivered via weekly self-administered inhalation, AR-501 achieved substantially higher pulmonary drug levels than earlier intravenous gallium formulations. In a completed Phase 2a trial in CF patients with confirmed *P. aeruginosa* infection (NCT03669614, *n* = 41), AR-501 met its primary and secondary endpoints and was well-tolerated across all dose levels. Most treatment emergent adverse events were respiratory in nature (e.g., cough and throat irritation), mild to moderate (grade 1–2) in severity and judged by the blinded investigator to be unrelated to the study drug; one grade 3 event (dry throat and cough) occurred after the first inhaled dose, and no drug-related serious adverse events were reported. Sputum gallium concentrations exceeded the level required to inhibit *P. aeruginosa* by more than 50-fold, as reported by the company [84]. Despite these Phase IIa results (announced in March 2023), no Phase IIb trial has been registered as of the most recent search. In December 2024, Aridis announced an asset acquisition agreement transferring exclusive ownership of AR-501 to an undisclosed pharmaceutical partner in exchange for upfront payments and future royalties [85]; the new owner’s development plans for the asset have not been publicly disclosed, leaving AR-501’s current clinical trajectory genuinely uncertain rather than confirmed to be discontinued.

## 3. Discussion

**Figure 2:**
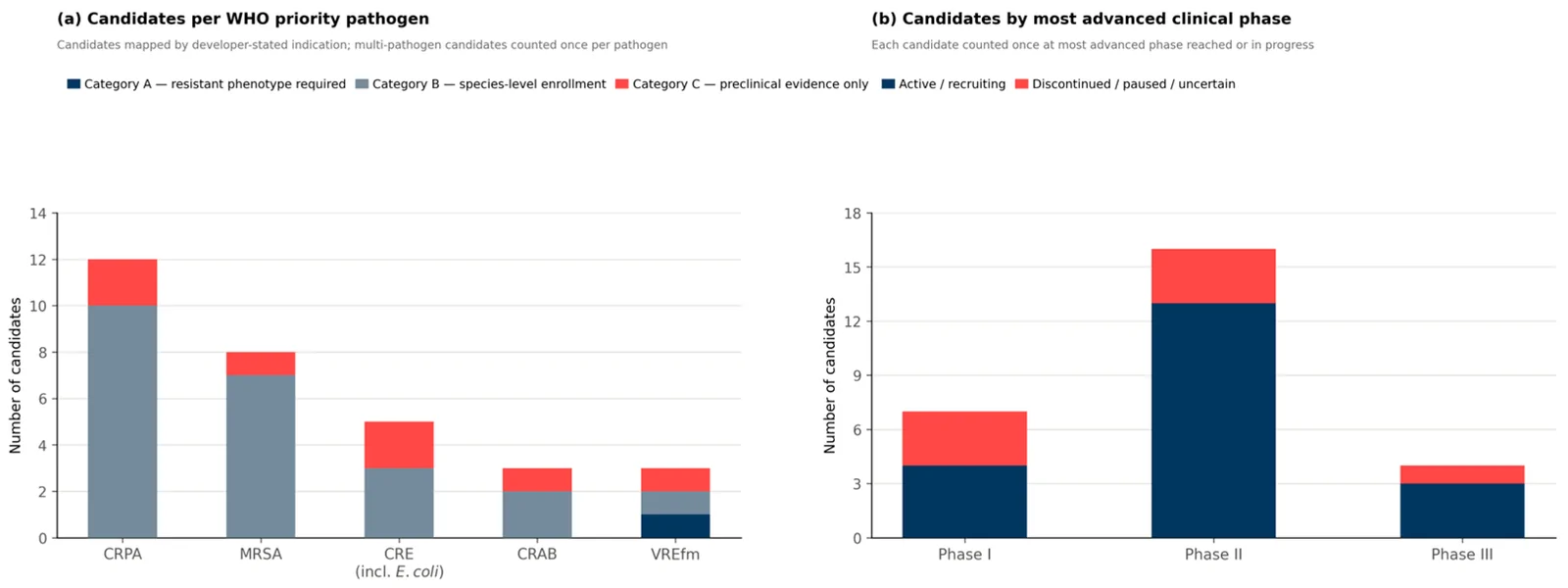
(**a**) Candidates per WHO priority pathogen; (**b**) candidates by most advanced clinical phase reached.

This review identified 27 non-traditional antibacterial candidates in clinical development across six broad modality classes—AMPs, bacteriophages, CRISPR-armed bacteriophages, LBPs, mAbs and small molecules/other—active in clinical development since January 2023. Twelve of the dataset candidates aim to target CRPA, eight aim to target MRSA, five aim to target CRE, three aim to target CRAB, and three aim to target VREfm, showing an unequal pathogen target distribution. Most candidates sit in early to mid-phase—phase II or equivalent—and only four out of the 27 candidates have reached phase III (Figure 2). While most candidates employ mechanisms that are theoretically independent of classical resistance genotypes, the majority of clinical programs enroll patients based on species-level colonization or infection rather than confirmed resistant phenotype, and explicit preclinical evidence against resistant isolates is inconsistently reported across the dataset.

### 3.1. The Translation Gap

A defining feature of the non-traditional antibacterial field (and the entire field in general) is the difficulty of going from early discovery to clinical testing—sometimes referred to as the antibiotic “Valley of Death”. This bottleneck describes the point at which preclinical candidates fail to advance into human trials, whether due to insufficient funding, unclear regulatory pathways for novel drug types, or the difficulty of translating in vitro or animal model efficacy into a clinical program [86].

The translation gap is evident from the WHO’s most recent preclinical pipeline review, which identified 232 antibacterial products in preclinical development worldwide as of February 2025 [87]. Approximately 40% (92) are non-traditional antibiotics (of which six target *M. tuberculosis*, and most of these non-traditional antibacterials are investigated by micro (43.5%) or small (33.7%) private companies or academic institutions (14.1%). Small molecules are the largest group, with 30 preclinical candidates, then comes bacteriophages with 22 candidates, large molecules with 15 candidates, antibodies with 7 candidates, and so on. Most of the products (55%) have an unknown pathogen target, whilst 45% of the preclinical candidates are pathogen-specific. Set against the comparatively small number of candidates that reach clinical-stage development—including the 27 active in this review—the difference illustrates how few preclinical programs ultimately progress to clinical trials.

This pattern is not new or specific to non-traditional approaches. Beyer and Paulin highlighted that the antibacterial clinical pipeline has long suffered from a low average success rate of ~14% and an average development time of 7 years from Phase I to approval. The WHO 2020 pipeline snapshot revealed a total of 60 antibacterials in the clinical pipeline compared to >5700 drug candidates in oncology [88]. Across all antibacterial therapeutics in development between 2011 and 2020, only 16.3% of Phase I antibacterial candidates gained FDA approval, and the last FDA-approved antibiotic with a genuinely novel mechanism of action against Gram-negative bacteria was discovered around 60 years ago. Prasad et al. notes that antibiotics developed in the 1980s–1990s had a much higher success rate than they do today, with ~40% reaching approval. The translation gap is thus only becoming larger over time [89].

### 3.2. An Uneven Pathogen Distribution

This review reveals a very clear uneven pathogen target distribution among the current non-traditional antibacterial clinical pipeline candidates. Twelve out of the 27 candidates target CRPA, eight target MRSA, five target CRE, three target CRAB, three target VREfm and one targets Shigella. There are quite a few non-traditional antibacterials targeting *M. tuberculosis*, which are not included in this review. So, only 7 out of the 15 bacterial priority pathogens have current non-traditional antibacterials in development for treatment.

The pathogens with the most clinical-stage activity (CRPA, MRSA) are predominantly hospital-acquired infections in well-resourced healthcare settings versus enteric/commensal pathogens (Shigella, Salmonella), which disproportionately burden low/middle-income countries, where trial logistics and commercial incentives are both weaker.

This pathogen-specific concentration is not static. As recently as 2021, CRAB and CRPA were jointly identified as the field’s most pressing unmet need, with critical priority pathogens collectively accounting for only a small fraction of the total clinical pipeline [10]. In the years since, clinical stage non-traditional development has shifted disproportionately toward CRPA, while CRAB-targeting candidates have not seen a comparable increase. This suggests that even within a recognized priority area, R&D investment does not track evenly with the stated unmet need.

Most candidates in the non-traditional pipeline are mapped to WHO priority pathogens by indication rather than by confirmed activity against the resistant phenotype specifically, consistent with how the broader pipeline literature treats pathogen mapping—but this also reflects a genuine gap in the public evidence base, since few trials are enrolling specifically on confirmed resistant phenotypes.

### 3.3. Mixed Clinical Maturity

Most of the clinical candidates identified in this review are in Phase I or II. Only two candidates have reached Phase III outright (Peceleganan, tosatoxumab), with two more in combined Phase II/III trials (PLG0206, LBP-EC01). The remaining 23 candidates sit in or were discontinued/paused in Phase I or Phase II, indicating that the field as a whole remains at an early stage of clinical maturity despite the span of modalities represented. Trial populations are also frequently narrow. Bacteriophages are the largest single-modality group in this review, and within it, CRPA-targeting candidates show a particularly pronounced pattern: four of five use inhaled delivery, predominantly restricted to CF populations—a very specific clinical niche relative to the broader burden of CRPA infection. Other candidates cluster around similar limited indications—TP-102 and BX211/BX011, for example, both specifically target diabetic foot infections. Also, there is a clear route-of-administration constraint when looking at the 27 candidates. A total of 10/27 candidates are tested via IV delivery versus 6 via oral delivery and 5 via inhaled delivery, with the remaining 6 split across topical, local, intra-articular and intraurethral routes. IV administration in particular limits usage to hospital setting, restricting reach into outpatient care and lower-resource settings where much of the global AMR burden is concentrated.

Taken together, these patterns suggest that route diversification and population breadth are not yet occurring in tandem: where a non-IV route does exist, as with inhaled bacteriophage therapy, it tends to come paired with a narrow population (CF specifically) rather than genuinely broadening access to a wider range of patients or settings.

### 3.4. Mixed Clinical Evidence

Beyond the pathogen- and healthcare-setting pattern described above, the clinical evidence within this pipeline itself is mixed. Several candidates have completed trials, with positive reported results. AP-SA02, a bacteriophage cocktail, met its efficacy endpoints in a Phase Ib/IIa trial for complicated S. aureus bacteremia, as reported in a conference abstract [34]. CMTX-101, a DNABII-targeting biofilm-disrupting mAb, achieved a greater than 70% reduction in *P. aeruginosa* CFU count by Day 28 in a completed Phase II cystic fibrosis trial [65]. RESP-X, an anti-PcrV mAb, met its primary objectives in a completed Phase IIa trial in non-CF bronchiectasis patients, as reported by the company [70]. The reduction in exacerbation rates was an exploratory secondary outcome and did not reach statistical significance [70].

By contrast, two mAbs targeting bacterial toxins and virulence factors directly—rather than disrupting biofilm structure—have struggled. Tosatoxumab, an anti-alpha-toxin antibody for S. aureus VAP, reached Phase III before stalling (now looking for a partnership to continue development) [71], while MEDI3902 (gremubamab), a bispecific antibody targeting the *P. aeruginosa* type III secretion system and Psl exopolysaccharide, was discontinued after failing to meet its primary endpoints in a randomized controlled trial of mechanically ventilated *P. aeruginosa*-colonized ICU patients. MEDI3902 was tested as a prophylactic strategy in this high-risk target population [73].

Notably, both groups include mAbs, which raises the possibility that mechanism, rather than modality, is the more relevant distinction. Antibodies engineered to disrupt biofilm architecture (CMTX-101, TLR1068) have so far produced more consistent positive signals than those designed to neutralize toxins or block adhesion directly (tosatoxumab, MEDI3902) and RESP-X, an anti-PcrV antibody evaluated in chronic colonization, has also reported positive early signals. This comparison is confounded, however, by clinical setting. The positive signals derive from chronic colonization or PJI, whereas the two failures occurred in acute VAP treatment or prophylaxis in mechanically ventilated ICU patients, and RESP-X and MEDI3902 share the PcrV target. Combined with AP-SA02’s success in a structurally unrelated modality (bacteriophage therapy), this suggests, but does not yet establish, that clinical progress may track more closely with specific mechanisms than with modality class. Given the small number of candidates, heterogeneous trial designs and non-comparable populations, these observations should be regarded as hypothesis-generating.

The available evidence level for each candidate was attributed to an evidence level 1–5, with Level 1 representing peer-reviewed trial data with a clinical endpoint and Level 5 representing preclinical or mechanistic support only (see Methods). Across the 27 candidates in this dataset, evidence levels vary. Only four candidates—PL-5, Tosatoxumab, MEDI3902 and CAL02—have peer-reviewed trial data with a clinical endpoint (Level 1) and of these, two (Tosatoxumab and MEDI3902) did not meet their primary endpoints in their initial indications. A further nine candidates have peer-reviewed early-phase trial data with microbiological endpoints (Level 2), while the remaining 14 candidates have their most advanced available evidence at the level of conference abstract, company press releases, or preclinical data (Levels 3–5).

A similar pattern is observed when considering pathogen indication specificity (see Methods): of the 27 candidates, only one (Category A) enrolls patients with the specific WHO-defined resistant phenotype (VRELysin, where VRE colonization was a confirmed enrollment criterion), while 19 enroll at the species level without requiring confirmed resistance (Category B) and six rely on preclinical or in vitro data to establish the link to the resistant pathogen (Category C)—with PLG0206 spanning both categories depending on the pathogen (Supplementary Table S1). Taken together, the evidence-level and indication-category distributions suggest that the non-traditional pipeline remains at an early stage of clinical validation against WHO priority pathogens and reflects the early-stage nature of the non-traditional pipeline overall.

### 3.5. The Paradox of Antibiotic Stewardship

This concentration of clinical-stage development on hospital-acquired infections in high-income settings, discussed above, reflects a broader disconnect. AMR does not operate in isolation. It intersects with climate change, malnutrition, infectious diseases, and socioeconomic disadvantage and adds to existing health gaps for already vulnerable populations. Pipeline investment—limited as it already is—does not closely track with where AMR’s burden falls hardest and the structural reasons for this misalignment go beyond simple underinvestment [2,90].

Several biotechnology companies—including Achaogen, Melinta Therapeutics and Entasis—have filed for bankruptcy in the years following the regulatory approval and market launch of their respective antibiotics [91], exposing a structural paradox at the heart of antibiotic stewardship. The prescribing restrictions required to preserve a new drug’s efficacy and delay resistance are the same restrictions that limit its sales volume, making commercial cost recovery exceptionally difficult even after a candidate successfully reaches the market. In effect, the pharmaceutical market remains poorly suited for short-course treatments of this kind, regardless of their clinical value, and the profit of developing new antibiotics remains too low even as the cost of not developing them rises.

In response to this funding gap and to help candidates cross the translation gap described earlier, several public–private partnerships and non-profit organizations have emerged specifically to support early-stage antibacterial development. CARB-X, a global non-profit partnership, funds and accelerates preclinical and early clinical antibacterial research globally, while the Global Antibiotic Research & Development Partnership (GARDP) focuses on developing and ensuring access to new treatments for the drug-resistant infections of greatest public health concern. Within this review’s own dataset, five candidates—PLG0206, TLR1068, CMTX-101, GSK3882347 and SER-155—have received CARB-X funding, while LBP-EC01’s later trials have been supported by BARDA.

GARDP points out that despite these efforts, new incentives are needed to address these major challenges, and that this will only occur if new national initiatives to repair the broken antibiotic market model are quickly implemented globally [92].

### 3.6. Resistance Risk in Non-Traditional Antibacterials: A Mechanism-Dependent Picture

A recurring rationale for non-traditional antibacterial development is that many of these mechanisms do not depend on inhibiting the same essential bacterial viability pathways targeted by traditional antibiotics, and so the classic single-mutation escape route of antibiotic resistance is less readily available. MacNair et al. frame this directly: the central argument for pursuing alternative therapeutic strategies is that traditional antibiotic discovery has been unable to keep pace with the rate at which resistance evolves, making mechanistically distinct approaches a necessary complement to the existing antibacterial arsenal [11].

The strongest direct empirical support outside this dataset comes from OligoG. Long-term in vitro exposure of *P. aeruginosa* biofilms to the oligomer over an extended culture period produced no extensive resistance-associated mutations and no enhanced cross-resistance to co-administered antibiotics—a finding consistent with its proposed mechanism of physical disruption of biofilm structure rather than inhibiting a single bacterial pathway, though phase IIb was terminated before January 2023 and no clinical progress has been reported since [93]. A second example, from the lysin field, reinforces this pattern. Serial-passage studies of exebacase found no change in minimal inhibitory concentration (MIC) against MRSA over 28 days, in contrast to the large MIC increases observed for comparator antibiotics tested in parallel [94]. Exebacase was nonetheless terminated in Phase III for clinical futility, not resistance, underscoring that a favorable resistance profile does not equal clinical success [95]. The available in vitro evidence is suggestive but limited. Both candidates providing the clearest resistance data—OligoG and exebacase—were discontinued before generating long-term clinical resistance data, meaning that any resistance-risk advantage of non-traditional mechanisms remains a theoretical prediction, supported by limited in vitro data, rather than an established clinical finding. This advantage is not likely to be uniform across modalities, and the available evidence suggests a more mechanism-specific picture rather than “non-traditional agents resist resistance”. Bacteriophage resistance via receptor mutation is documented and does occur in practice, but resistance often imposes a measurable fitness cost on the bacterium—for example, it reduces capsule formation, which increases susceptibility to host immune clearance. Notably, some phages specifically target efflux pumps. Gaining resistance to these pumps can re-sensitize the pathogen to antibiotics it has previously been resistant to [33]. mAbs present a different risk profile. Their narrow pathogen- or epitope-specific targeting avoids the broad selective pressure on commensal flora that drives much conventional antibiotic resistance [96]. Antigenic escape, where mutation or downregulation of the target epitope itself occurs, is a real risk of this modality.

Together, this is consistent with the pattern observed earlier. Clinical success and resistance risk within the non-traditional space appear to track more closely with specific mechanism than with broad modality. Non-traditional antibacterials may exhibit mechanism-dependent differences in the propensity for resistance development, but clinical evidence remains insufficient to establish a consistently lower resistance risk. What the available data support is that resistance risk within this space is mechanism-specific rather than uniformly lower and should be understood as a different risk profile rather than the absence of risk.

### 3.7. Limitations

This review has several limitations. Candidate identification and categorization were performed by a single reviewer, without independent duplicate screening. This may have introduced selection or extraction errors, which a second independent reviewer would otherwise have caught. No formal risk-of-bias or quality assessment was applied to the included clinical evidence, which varies considerably in trial size and design. Efficacy signals from company press releases and conference abstracts should therefore be interpreted with caution relative to peer-reviewed randomized trial data. Comparisons drawn across candidates in this review should be read as descriptive rather than as a quality-weighted ranking of evidence.

The classification of AMPs as non-traditional agents follows the framework proposed by Butler et al. [10] and MacNair et al. [11], rather than other frameworks that classify them as traditional agents. *M. tuberculosis* was excluded given its distinct disease mechanisms and membrane structure, but a substantial non-traditional pipeline exists for this pathogen and is not captured here. Finally, candidate status was determined from ClinicalTrials.gov, peer-reviewed publications, and company communications current as of 24 June 2026—given the pace of change, some candidates’ development status might have shifted between data collection and publication.

### 3.8. Future Research Directions

Antibacterial stewardship is fundamental to controlling AMR, but it cannot stand alone nor can it fix the underlying market failure driving the translation gap described above. There is an urgent unmet need for economic incentives to bridge the financial gap between antibiotic R&D costs and antibiotic sales revenue. In 2019 the UK announced a national action plan to investigate a trial payment model based on medicinal value to the health system, de-linking payments from the volume of antibiotics sold. NHS England and NICE have described the pilot program as successful and it was approved for full implementation in 2024 [97]. More broadly, Anderson et al. evaluated four different incentive options: subscription payments, market entry rewards, transferable exclusivity extensions, and milestone payments—concluding that no single incentive can work alone. Each has distinct strengths and weaknesses and the incentive design matters as much as incentive size [98].

Alongside financing reform, priority should be given to agents whose mechanisms are expected, on mechanistic grounds, to reduce selection pressure for resistance, while recognizing that this expectation remains to be confirmed clinically. Pathogens can acquire resistance to a new antibiotic alarmingly fast once they reach the market, in effect forming an ongoing arms race between bacteria and traditional antibiotics. Non-traditional approaches that avoid the same pathways and proteins targeted by conventional antibiotics may offer one route to circumvent this, although this remains to be demonstrated in long-term clinical use.

Greater focus is also needed on the neglected bacterial priority pathogens. *Shigella*, a high-priority pathogen, with only one non-traditional antibacterial in clinical development, is ranked among the leading causes of diarrheal disease in children under five years of age [99]. The consequences of this neglect are very visible in high-burden settings: in Bangladesh, fluoroquinolone resistance prevalence among *Shigella* isolates reached 61.9%, disproportionately affecting young children and impoverished populations [100].

Beyond financing and pathogen prioritization, structural barriers can stall even scientifically promising candidates. DSTA4637S, developed by Genentech, illustrates this well. Excluded from this review’s primary dataset, it is an antibody–antibiotic conjugate designed to deliver a rifamycin-class antibiotic (dmDNA31) directly into phagocytes, via a protease-cleavable linker attached to an anti-*S. aureus* IgG1 mAb [101]. The rationale addresses a genuine unmet need. Intracellular persistence is one of the main reasons that *S. aureus* bacteremia is so difficult to cure. Yet despite promising safety data (NCT03162250), DSTA4637S has not advanced beyond Phase Ib as of the most recent available information. This is an example of the economic and development barriers facing novel non-traditional approaches.

One further modality sits outside this review’s scope. Bacteriophage-derived lysins are mechanistically related to the bacteriophage candidates already covered here. These enzymes directly degrade bacterial cell wall peptidoglycan, offering rapid bactericidal activity, a reportedly low tendency for resistance development in vitro and high target specificity. No lysins have been in active development after January 2023 and they are thus not included in this review [94]. Engineering advances such as domain recombination and antimicrobial peptide fusion have begun extending lysin activity further—for example, the Artilysin Art-175, which combines a lysin with a membrane-permeabilizing peptide, has shown efficacy against resistant *A. baumannii* [102]. Another candidate, exebacase, whose favorable in vitro resistance profile was noted above, nonetheless illustrates that theoretical promise does not guarantee clinical success. Despite reaching Phase III and receiving FDA Breakthrough Therapy designation based on its encouraging Phase II results (NCT03163446), its DISRUPT trial (NCT04160468) was terminated early for futility in MRSA bacteremia and endocarditis [95].

Future clinical trials should, where feasible, enroll patients with confirmed resistant phenotype rather than species-level colonization status and trial design should include comparators that allow meaningful cross-candidate efficacy comparisons.

## 4. Methods

Candidates for inclusion were identified from the WHO “Antibacterial products in clinical development for bacterial priority pathogens” (published in October 2025), which categorizes agents in clinical development as traditional or non-traditional. From this report’s non-traditional category, candidates were retained if their indication included at least one WHO priority pathogen. Supplementary searches of ClinicalTrials.gov, the Global AMR R&D Hub, ISRCTN and company pipeline pages were conducted using candidate names and the terms ‘non-traditional antibacterial’, ‘bacteriophage’, ‘antimicrobial peptide’, ‘monoclonal antibody AMR’, in combination with WHO priority pathogen names. The last search was conducted on the 24th of June 2026. *M. tuberculosis* was excluded from the analysis, as, despite being a WHO priority pathogen, it is conventionally treated as a separate field given its distinct disease mechanism and membrane structure relative to the other priority pathogens.

Candidates were included if they targeted a bacterial species associated with a WHO priority pathogen (e.g., *P. aeruginosa* for CRPA, *S. aureus* for MRSA), as indicated by company indications, rather than requiring confirmed activity against the specific resistant phenotype in every case. For most candidates, the available clinical and preclinical evidence demonstrates broad activity against the relevant species, including isolates carrying the resistance mechanism defining the WHO priority pathogen category (e.g., methicillin resistance for MRSA), but in several cases trial populations were defined by clinical syndrome or species-level colonization status rather than confirmed resistant phenotype of the infecting isolate. Where this distinction is relevant to interpreting a candidate’s evidence base, it is noted in the corresponding Results entry.

For each retained candidate, development status was verified and updated using the Global AMR R&D Hub—which aggregates data from the WHO pipeline reviews and the now-discontinued PEW Charitable Trusts antibiotic tracker (discontinued 2021)—together with ClinicalTrials.gov and targeted searches for peer-reviewed updates or press releases reporting on each candidate’s status. This was performed by searching for the name of the antibacterials and if a candidate had multiple names, separate searches for both were performed. Candidates were included if this verification process identified evidence of clinical activity—a trial start, primary completion, or last-update on ClinicalTrials.gov; a peer-reviewed publication or conference abstract reporting clinical data; a company communication confirming development status—dated January 2023 or later. Peer-reviewed publications, conference abstracts, and company communications (press releases and pipeline updates) were weighted equally for the purpose of establishing whether a candidate met this activity threshold. No source type was treated as a stronger or weaker indicator of active development than another for inclusion purposes. Where multiple sources reported conflicting statuses for the same candidate, the most recently dated source was used to determine the current status. For the purpose of evaluating efficacy and safety claims, by contrast, evidence was weighted by source type: published randomized controlled trials and published non-randomized clinical studies represent the strongest evidence, followed by conference abstracts reporting clinical data, followed by unpublished company communications. This hierarchy is reflected in the evidence level framework (L1–L5) described below and in the source attributions used throughout the Result sections. Because the L1–L5 framework grades evidence by source type and endpoint rather than by study design, randomization status is stated explicitly in the individual candidate entries. Activity status was defined as follows: *discontinued*—development has been reported to have ceased; *paused*—development is not currently active but is reported or expected to resume at a later time point, including pauses attributed to funding; *uncertain*—no recent update on development status could be identified.

This decision reflects the review’s aim of characterizing not only which candidates remain active in development, but also where and why candidates have left the pipeline since January 2023—information considered relevant to assessing the overall non-traditional antibacterial field. Candidates with no documented activity of any kind after January 2023 were excluded entirely. 

The available evidence level for each candidate was attributed one of five evidence levels: Level 1: peer-reviewed trial with a clinical endpoint; Level 2: peer-reviewed early-phase clinical trial with a microbiological endpoint; Level 3: conference abstracts or trial registry result only; Level 4: company press release or pipeline update only; Level 5: preclinical or mechanistic data only, with no published clinical evidence. Where a candidate had evidence from multiple source types, the highest applicable level was assigned. Evidence level assignments are provided in Supplementary Table S1 and reflect the strongest publicly available evidence as of the last search date (24 June 2026). Pathogen indication category (A, B or C) is also provided in Supplementary Table S1: Category A—trial enrollment requires the specific WHO-defined resistant phenotype; Category B—trial enrolls at the species level without requiring the resistant phenotype at enrollment; Category C—the link to the resistant pathogen is supported by preclinical or in vitro data only, and enrolled patients in completed or ongoing trials were not required to have the specific resistant infection (including healthy volunteer Phase I trials).

Candidate identification, status verification and categorization were performed by a single reviewer. 

Antimicrobial peptides (AMPs) were classified as non-traditional antibacterial agents consistent with the frameworks proposed by Butler et al. [10]. and MacNair et al. [11]. This classification reflects their peptide nature, biological origin and mechanisms of action via physical membrane disruption rather than target-specific biochemical inhibition. This contrasts with alternative frameworks (e.g., Melchiorri et al.) [103] that classify AMPs as traditional agents on the basis of their direct bactericidal activity. Non-traditional antibacterials in clinical development were categorized into the following groups: AMPs; bacteriophages targeting MRSA; bacteriophages targeting CRPA; polymicrobial bacteriophages; CRISPR-armed bacteriophages targeting *E. coli*; live biotherapeutic products; monoclonal antibodies targeting biofilm disruption; anti-virulence/anti-toxin monoclonal antibodies; anti-virulence small molecules and other agents. Applying these criteria identified a total of 27 non-traditional antibacterial candidates with documented activity against WHO priority pathogens since January 2023, of which 20 remain in active clinical development and seven have an unverified status or have since been discontinued, paused or halted. These are presented by modality. Individual candidate status is indicated in Table 1. Three candidates (PL-5, TP-102, MEDI3902) were identified through supplementary searches of ClinicalTrials.gov, national trial registries (ISRCTN), and company pipeline pages that were not captured in the WHO October 2025 non-traditional category, either due to classification differences or registry coverage gaps. OMN6 was identified in the WHO report but classified under the traditional category; it was reclassified as non-traditional in this review, consistent with the AMP framework applied throughout. MEDI3902 was first identified as discontinued but the development status was updated following identification of the GREAT-2 trial results (ISRCTN70034823) through targeted registry searches conducted after the WHO October 2025 report cutoff.

During manuscript preparation, Claude (Anthropic, claude.ai, Sonnet 4.6) was used as a research assistance tool for source verification, literature searching, and structural feedback. All scientific content, candidate selection, data interpretation, and conclusions were determined and verified by the authors.

## 5. Conclusions

Antimicrobial resistance remains a growing and urgent global threat, responsible for a substantial and rising burden of death and morbidity each year. Resistance has emerged against most traditional antibiotics currently in clinical use and the search for non-traditional approaches—agents that act through mechanisms distinct from traditional antibiotics—has accordingly become an increasingly active, if still comparatively small, area of antibacterial development.

This review identified 27 non-traditional antibacterial candidates with documented clinical activity since January 2023, of which 20 remain in active development and seven have been discontinued, paused or have unverified status. The pipeline is dominated by bacteriophages and is thin relative to the scale of the threat it aims to address, and its candidates are unevenly distributed across the WHO priority pathogen list. Of the 27 candidates identified, 12 target CRPA and eight target MRSA, while CRAB, CRE and VREfm are each targeted by three or fewer candidates and no non-traditional candidate in clinical development targets the *Salmonella* group. Of the 27 candidates, only four have reached Phase III, and the majority address chronic colonization or prophylaxis rather than established drug-resistant infection. Given the long and costly path from Phase I to market, and the well-documented translational gap between preclinical promise and clinical success, this concentration leaves much of the WHO priority pathogen list without a non-traditional alternative on the near-term horizon. This imbalance persists and is likely made worse by the chronic underinvestment in the field. Public and philanthropic funding for antibacterial R&D totaled only $2.51 billion across 2017 and 2023, a seven-year total against an estimated $251–276 billion in global pharmaceutical R&D spending in a single recent year.

Beyond cataloging the pipeline, this review highlights that most positive signals in the non-traditional space remain at the level of early-phase or company-reported evidence, and that the clinical setting—chronic colonization versus acute infection—may matter as much as modality in determining which candidates are most likely to reach the market.

Despite these gaps, non-traditional antibacterials represent a genuinely promising and likely necessary component of the broader response to AMR, rather than a replacement for traditional antibiotic development. Realizing their potential across the full WHO priority pathogen list will require sustained investment and a more deliberate effort to direct non-traditional pipeline activity toward the pathogens with the greatest unmet need.

## Figures and Tables

**Table 1:** Non-traditional antibacterial agents in clinical development. WHO Bacterial Priority Pathogen List 2024—candidates identified with clinical activity since January 2023. Candidates are grouped by modality (row background color and section headers). Filled circles (●) indicate developer-stated pathogen indications, mapped per the WHO antibacterial pipeline report (October 2025). and ClinicalTrials.gov; this does not imply confirmed activity against the specific resistant phenotype in all cases. Status: ● green = active/recruiting; ● red = stalled/discontinued/terminated; ● purple = status unverified as of the last search (24 June 2026). Italic species names in the “Other pathogen” column indicates candidates with indications outside the five WHO priority pathogens in the existing columns. Bracketed numbers correspond to footnotes below the figure. Abbreviations: i.a. = intra-articular; i.u. = intraurethral; IV = intravenous; CRAB = carbapenem-resistant *Acinetobacter baumannii*; CRPA = carbapenem-resistant *Pseudomonas aeruginosa*; CRE = carbapenem-resistant *Enterobacterales*; MRSA = methicillin-resistant *Staphylococcus aureus*; VREfm = vancomycin-resistant *Enterococcus faecium*. The Cat. column indicates the pathogen indication: category: A = enrollment restricted to WHO-defined resistant phenotype; B = species-level enrollment without a resistance phenotype requirement; C = preclinical/in vitro link only.

Asset/Drug (Synonym)	Phase	Route	Developer	CRAB	CRE	CRPA	MRSA	VREfm	Cat.	Other Pathogen	Note
**Antimicrobial peptides (AMPs)**											
● Peceleganan (PL-5)	III	Topical	Jiangsu ProteLight						**C**		†1
● PLG0206	II/III	i.a.	Peptilogics	●	●	●	●	●	**B/C**		
● OMN6	II	IV	OMNIX Medical	●					**B**		
**Bacteriophages—MRSA**											
● AP-SA02	Ib/IIa	IV	Armata Pharmaceuticals				●		**B**		†2
● BX011 (former BX211)	II	Local	BiomX				●		**B**		†3
● PP1493 + PP1815	II	i.a.	Phagenix (f. PHAXIAM)				●		**B**		†4
**Bacteriophages—CRPA**											
● AP-PA02	IIb	Inhaled	Armata Pharmaceuticals			●			**B**		†2
● BX004-A	Ib/IIa	Inhaled	BiomX			●			**B**		†5
● YPT-01	Ib/II	Inhaled	Yale University			●			**B**		
● WRAIR-PAM-CF1	Ib/II	IV	NIAID			●			**B**		†10
● TP-122A	I/IIa	Inhaled	TechnoPhage			●			**B**		
**Bacteriophages—polymicrobial**											
● TP-102	IIb	Topical	TechnoPhage	●		●	●		**B**		†3
**CRISPR-armed bacteriophages—*E. coli***											
● LBP-EC01	II/III	IV+i.u.	Locus Biosciences		●				**B**	*E. coli*	
● SNIPR-001	Ib	Oral	SNIPR Biome		●				**B**	*E. coli*	
**Live biotherapeutic products**											
● VRELysin	I/IIa	Oral	Intralytix					●	**A**		†7
● SER-155	Ib	Oral	Seres Therapeutics		●			●	**B**		
**mAb—biofilm disrupting**											
● CMTX-101	Ib/IIa	IV	Clarametyx Biosciences			●			**B**		†6
● TLR1068	II	IV	Trellis Bioscience				●		**B**		†6
● RESP-X (INFEX702)	IIa	IV	Infex Therapeutics			●			**B**		
**mAb—anti-virulence/anti-toxin**											
● Tosatoxumab (AR-301)	III	IV	Aridis Pharmaceuticals				●		**B**		[7]
● MEDI3902 (Gremubamab)	II	IV	AstraZeneca			●			**B**	NCFB	[8]
**Anti-virulence small molecules/other**											
● CAL02	II	IV	Eagle Pharmaceuticals						**C**	Pneumonia	
● AR-501/Panaecin	IIa	Inhaled	Aridis Pharmaceuticals			●			**B**		†9
● Rhu-pGSN	IIb	IV	BioAegis Therapeutics			●			**C**	ARDS	†8
● GSK3882347	Ib	Oral	GlaxoSmithKline		●				**C**	*E. coli*	
● ALS-4	I	Oral	Aptorum				●		**C**		
**Pipeline gaps—neglected pathogens**											
● ShigActive	I/IIa	Oral	Intralytix						**C**	*Shigella* spp.	

†1 Published Phase III data (JAMA Netw Open 2024); registered as Phase II on ClinicalTrials.gov. †2 Phase III planned for initiation 2026. †3 Development path transitioned to fixed cocktail; Phase II/III planned, not yet registered. †4 PHAXIAM Therapeutics entered judicial liquidation Sept 2025; assets acquired by Phagenix. †5 Phase IIb discontinued December 2025 following safety review. †6 Shares DNABII biofilm-disruption mechanism (CMTX-101 and TLR1068). †7 Phase IIa paused due to lack of funding. †8 EVADE trial (VAP prophylaxis) failed primary endpoint; GREAT-2 trial (bronchiectasis, chronic Pa) met primary endpoint (ATS/ERS 2025). AZD0292 (half-life extended version) in development. †9 Ownership transferred to undisclosed partner December 2024; future development plans unknown. †10, IV delivery; no results published as of most recent search; italic species names in ‘Other pathogen’ column indicate indications outside the five core WHO priority pathogens.

## Data Availability

No new primary data were generated in this study. All data supporting the findings of this review are derived from publicly available sources including the WHO antibacterial clinical pipeline report (October 2025), ClinicalTrials.gov, the Global AMR R&D Hub, peer-reviewed publications, conference abstracts, and company communications, as cited in the reference list. A full candidate-level data summary is provided in Table S1 (Supplementary Materials).

## Supplementary Materials

The following supporting information can be downloaded at: https://www.mdpi.com/article/10.3390/antibiotics15090899/s1, Table S1: Full candidate-level summary of non-traditional antibacterial agents included in this review. References cited in Supplementary Table S1 include [30,46].

## Abbreviations

The following abbreviations are used in this manuscript:

AMR	Antimicrobial resistance
AMP	Antimicrobial peptide
BARDA	Biomedical Advanced Research and Development Authority
BPPL	Bacterial Priority Pathogen List
BSI	Bloodstream infection
CRAB	Carbapenem-resistant *Acinetobacter baumannii*
CRE	Carbapenem-resistant Enterobacterales
CRPA	Carbapenem-resistant *Pseudomonas aeruginosa*
CRISPR	Clustered Regularly Interspaced Short Palindromic Repeats
CF	Cystic fibrosis
CFU	Colony-forming unit
DAIR	Debridement, Antibiotics and Implant Retention
DFO	Diabetic foot osteomyelitis
DFU	Diabetic foot ulcer
FQR	Fluoroquinolone-resistant
GI	Gastrointestinal
HSCT	Hematopoietic stem cell transplant
i.a.	Intra-articular
ICU	Intensive care unit
i.u.	Intraurethral
IV	Intravenous
LBP	Live biotherapeutic product
mAb	Monoclonal antibody
MDR	Multidrug-resistant
MIC	Minimal inhibitory concentration
MoA	Mechanism of action
MRSA	Methicillin-resistant *Staphylococcus*
NCFB	Non-cystic fibrosis bronchiectasis
PJI	Prosthetic joint infection
PK	Pharmacokinetics
RCT	Randomized controlled trial
ROS	Reactive oxygen species
SAD/MAD	Single/multiple ascending dose
SOC	Standard of care
UTI	Urinary tract infection
VAP	Ventilator-associated pneumonia
VREfm	Vancomycin-resistant *Enterococcus faecium*
WHO	World Health Organization
3GC-RE	Third-generation cephalosporin-resistant Enterobacterales
